# Mesenchymal stem cells accelerated growth and metastasis of neuroblastoma and preferentially homed towards both primary and metastatic loci in orthotopic neuroblastoma model

**DOI:** 10.1186/s12885-021-08090-2

**Published:** 2021-04-10

**Authors:** Jiao-Le Yu, Shing Chan, Marcus Kwong-Lam Fung, Godfrey Chi-Fung Chan

**Affiliations:** 1 Hematology Oncology Center, Beijing Children’s Hospital, Capital Medical University, National Center for Children’s Health, Beijing, China; 2 Department of Paediatrics and Adolescent Medicine, Li Ka Shing Faculty of Medicine, The University of Hong Kong, Hong Kong, Special Administrative Region, China; 3 Department of Paediatrics and Adolescent Medicine, Hong Kong Children’s Hospital, Hong Kong, Special Administrative Region, China

**Keywords:** Mesenchymal stem cells, Neuroblastoma, Growth, Metastasis, Homing

## Abstract

**Background:**

Majority of neuroblastoma patients develop metastatic disease at diagnosis and their prognosis is poor with current therapeutic approach. Major challenges are how to tackle the mechanisms responsible for tumorigenesis and metastasis. Human mesenchymal stem cells (hMSCs) may be actively involved in the constitution of cancer microenvironment.

**Methods:**

An orthotopic neuroblastoma murine model was utilized to mimic the clinical scenario. Human neuroblastoma cell line SK-N-LP was transfected with luciferase gene, which were inoculated with/without hMSCs into the adrenal area of SCID-beige mice. The growth and metastasis of neuroblastoma was observed by using Xenogen IVIS 100 in vivo imaging and evaluating gross tumors ex vivo. The homing of hMSCs towards tumor was analyzed by tracing fluorescence signal tagged on hMSCs using CRI Maestro™ imaging system.

**Results:**

hMSCs mixed with neuroblastoma cells significantly accelerated tumor growth and apparently enhanced metastasis of neuroblastoma in vivo. hMSCs could be recruited by primary tumor and also become part of the tumor microenvironment in the metastatic lesion. The metastatic potential was consistently reduced in lung and tumor when hMSCs were pre-treated with stromal cell derived factor-1 (SDF-1) blocker, AMD3100, suggesting that the SDF-1/CXCR4 axis was one of the prime movers in the metastatic process.

**Conclusions:**

hMSCs accelerated and facilitated tumor formation, growth and metastasis. Furthermore, the homing propensity of hMSCs towards both primary tumor and metastatic loci can also provide new therapeutic insights in utilizing bio-engineered hMSCs as vehicles for targeted anti-cancer therapy.

**Supplementary Information:**

The online version contains supplementary material available at 10.1186/s12885-021-08090-2.

## Background

Neuroblastoma is the most common extra-cranial solid neoplasm in children which accounting for 7–10% of all pediatric tumors, in particularly for children less than one year of age. The incidence of neuroblastoma is 1 in 7000 live births and 96% of patients are younger than 10 years old [1]. Except a few of those who undergo spontaneous regression during the infancy period, around 65% of patients have metastatic disease at diagnosis, and they are sensitive to chemotherapy but tend to recur. Despite multimodality therapeutic approaches, most of them have either refractory disease or relapse and the outcome of high-risk neuroblastoma is far from satisfactory even with current therapeutic approaches [2, 3]. How to improve the prognosis of aggressive neuroblastoma remains a major challenge.

The current issue is how to tackle the underlying mechanisms responsible for tumorigenesis and metastasis. The growth and progression of cancer cells require the support from the surrounding microenvironment. Mesenchymal stem cells (or mesenchymal stromal cells, MSCs) have been postulated to be actively involved in the constitution of cancer microenvironment and is responsible for tumorigenesis, metastasis and immune evasion. MSCs are multi-potent somatic stem cells. The capability of multi-lineage differentiation and distinct immunomodulatory properties propel MSCs as one of the favorite therapeutic cells of choice particularly for tissue regeneration. Paradoxically, such capacities also facilitate tumor cell survival.

The relationship between MSCs and cancer has been considered to be a dual and interactive balance. This fact is reflected by two of the following evidences. On one hand, MSCs can preferentially migrate towards tumor and thus take part in tumor growth, vice versa, cancer cells can be preferentially attracted by MSCs residing in bone marrow leading to bony metastasis. Even more importantly, MSCs can provide pro-tumorigenic sanctuary for tumor growth and metastasis. The potential mechanisms underlying “cancer-friendly” effects of MSCs may involve: (1) contributing to pro-cancer microenvironment by differentiating into cancer-associated cellular components including fibroblasts, pericytes and other connective tissues; (2) enhancing neo-vascularization through secreting neurogenic and angiogenic factors (e.g. vascular endothelial growth factor); (3) producing growth factors promoting cancer growth; (4) releasing soluble factors to enhance distant metastasis particularly bone metastasis and increasing the survival of cancer cells; (5) regulating the immune system to favor cancer development [4–7]. Although majority of studies support MSCs can accelerate the growth and progression of tumor [5, 8–10], however, anti-cancer effects were also observed in other studies [11–13]. So far, the impact of MSCs in cancer microenvironment remains controversial. It has been indicated that the survival of neuroblastoma could be supported by MSCs in some in vitro studies [14, 15]. Our previous study has demonstrated that MSCs could enhance the metastasis of neuroblastoma via stromal cell derived factor-1 (SDF-1) signaling pathway in vitro [16]. However, conflicting results was also reported [17]. Underlying mechanisms attributed to the conflicting research results are potentially dependence on the particular research design, different in vitro culture conditions and specific tumor model. It has been argued whether the current tumor model exactly reflects the natural microenvironment since majority of studies established animal tumor model by subcutaneous transplantation. A suitable orthotopic preclinical model is required to mimic the in vivo tumor microenvironment scenario. We therefore aim to explore this dynamic relationship by performing cellular mixing experiments and co-implant MSCs with neuroblastoma cells in vivo.

In this study, an orthotopic murine model was adopted to investigate the impact of MSCs on tumorigenesis and metastasis of neuroblastoma. Homing property and the underlying mechanisms was further explored.

## Methods

### Materials and reagents

AMD3100, a specific antagonist of Stromal cell-derived factor-1α (SDF-1α)‘s receptor CXCR4, was purchased from Sigma-Aldrich (St. Louis, MO, USA). Lipofectamine® 2000 was from Invitrogen (Carlsbad, CA, USA). Lipophilic fluorescence dye-CM DiI was from Molecular Probes (Carlsbad, CA, USA).

### Cell culture

The bone marrow human MSCs (hMSCs) were isolated from healthy bone marrow transplantation donors by density-gradient centrifugation with Ficoll-Hypaque (GE Healthcare, Little Chalfont, UK). Written informed consent was obtained under the approval of the Combined Internal Review Board (Ethical Committee) of the University of Hong Kong and The Hong Kong West Cluster of Hospital Authority. The immunophenotype and differentiation characteristics of hMSCs were clarified by surface marker definition and differentiation assays. The homogenous hTertMSCs, an immortalized hMSCs cell line with human telomerase reverse transcriptase gene inserted, was a gift from Prof. D. Campana (St Jude Children’s Research Hospital, Memphis, TN, USA) [18]. All hMSCs were cultured in vitro with Dulbecco’s Modified Eagles Medium-low glucose (DMEM-LG; GIBCO, Invitrogen, Carlsbad, CA, USA) supplemented with 10% fetal bovine serum (FBS), 100 U/mL penicillin, 100 mg/mL streptomycin and 2 mM L-Glutamine.

Human neuroblastoma cell line SK-N-LP (a gift from Prof. NK Cheung, Memorial Sloan-Kettering Cancer Centre, NY, USA) was cultured with Dulbecco’s Modified Eagles Medium-high glucose (DMEM-HG, Invitrogen, Carlsbad, CA, USA) at 37 °C supplemented with 10% FBS, 100 U/mL penicillin, 100 mg/mL streptomycin and 2 mM L-Glutamine.

### Cell labeling

hMSCs were pre-labeled with the lipophilic fluorescence dye-CM DiI (Molecular Probes, Carlsbad, CA, USA) before in vivo transplantation. In brief, cells were washed and incubated with CM-DiI at concentration of 5 μl/mL for 20 min at 37 °C and then washed three times with normal growth medium according to the instructions of the manufacturer. The concentration and incubation period were optimized by series of tests. The labeling efficiency was detected to be more than 99% without cytotoxicity and the strong fluorescence signal has been proven to be persistent for more than one month.

### Cell transfection and culture of bioluminescent human neuroblastoma cell line

Human neuroblastoma cell line SK-N-LP were transfected with plasmid expressing luciferase (a kind gift from Prof. Nancy Kwan Man, HKU) using Lipofectamine® 2000 (Invitrogen, Carlsbad, CA, USA) following the manufacturer’s instructions. In brief, cells were cultured in 6-well plate and allowed to grow until they were 70–80% confluent. Plasmid DNA was diluted with DMEM without FBS and mixed with prepared Lipofectamine® 2000 solution. The mixture was added into cells and cultured at 37 °C overnight. Stable cell line was obtained by neomycin (G418) selection. The bioluminescence of luciferase gene-transfected cells was confirmed under Xenogen IVIS 100 imaging system. Luciferase gene-transfected SK-N-LP cells were culture with DMEM-HG supplemented with 10% FBS at 37 °C and sub-cultured when growing to 70–80% confluence.

### Orthotopic neuroblastoma model and xenografts of human luciferase-SK-N-LP cells

This in vivo project obtained the approval of Hong Kong Department of Health and Committee on the Use of Live Animals in Teaching and Research (CULATR), The University of Hong Kong. All procedures and animal care were under the surveillance of the committee. 6-week severe combined immunodeficiency (SCID)-beige mice (18.73 ± 0.76 g) were obtained from the Laboratory Animal Unit, The University of Hong Kong and were housed at specific pathogen-free facility with temperature of 22 ± 1 °C, humidity of 55 ± 5% and bred with autoclaved food and water ad libitum. Animals were monitored twice daily and initial number of mice in each test group was limited to 5 as required by the CULATR.

Luciferase-SK-N-LP cells were trypsinized from the culture flasks and prepared into single cell suspension. Cell viability was analyzed to be more than 99% using trypan blue exclusion assay. The single cell suspension at a concentration of 2×10^7^/mL and 0.2×10^6^ cells diluted with10μl 50% matrigel (BD Bioscience, Bedford, MA, USA) were prepared in equal volume and maintained on ice. The mixture of SK-N-LP and hMSCs was prepared by mixing SK-N-LP and hMSCs in ratio of 2:1. The final injected number of SK-N-LP cells was 0.2×10^6^.

Animals were anesthetized by intra-peritoneal injection of 100 mg/kg pentobarbital. After the disinfection with alcohol and betadine, an incision was made vertically in the abdomen of anesthetized SCID-beige mice. Left kidney was exposed gently and 20 μl of cell mixture was slowly injected into the fat pad of the adrenal gland adjacent to the upper pole of left kidney. The organs were carefully rearranged back and the incision was closed. The whole surgery was performed under strict aseptic technique to avoid infection. Mice were monitored until regaining consciousness. During the first 3 days post-surgery, the mice were given meloxicam in drinking water to minimize the pain at the dose of 0.3 mg/kg.

Following the intraperitoneal injection with D-luciferin (Gold Biotechnology, St Louis, MO, USA), the bioluminescence of transplanted cells of SK-N-LP group (*n* = 4) and hMSCs co-transplantation group (*n* = 4) was compared by Xenogen IVIS 100 in vivo imaging which was used to evaluate the in vivo initiation and progression of neuroblastoma. Signal intensity of regions of interest was analyzed by Living Image® Software (Xenogen, corporation Alameda, CA). Study designed for exploring the role of hMSCs in the growth and metastasis of neuroblastoma was shown in Supplementary Figure 1.

### Treatment and transplantation of hMSCs

CM DiI pre-labeled hMSCs (1 × 10^6^) were cultured with phosphate buffered saline (PBS) or specific CXCR4 antagonist AMD3100 (10 μg/mL) in suspension for 1 h, respectively. Then they were intravenously injected into mice with implanted neuroblastoma 7 weeks post-surgery via tail vein (*n* = 4). Before injection, the staining efficiency and cell viability were evaluated. Experimental design for exploring the tumor tropism property of hMSCs towards primary tumor and metastatic loci was illustrated in Supplementary Figure 1.

### Tumor volume evaluation

Three dimensions of isolated tumors were measured using a digital caliper and the volume was calculated according to the following formula. Tumor volume = Length×Wideth×Height× 1/2.

### Tumor metastatic loci detection

Mice were sacrificed by an overdose of pentobarbital. Organs including brain, lung, heart, liver, spleen, gut and bone were harvested immediately and washed twice with PBS. The metastatic loci of neuroblastoma were detected using Xenogen IVIS 100 imaging.

### Evaluation of hMSCs trafficking in vivo

The trafficking of hMSCs in vivo was traced using CRI Maestro™ imaging system by detecting the fluorescence signal of CM-DiI pre-labeled hMSCs in freshly harvested tumors and organs. Integrated density of regions of interest was analyzed by ImageJ (National Institutes of Health, USA).

### Statistical analysis

Comparison between means from different groups was analyzed using unpaired one-tailed Student *t* test for tumor volume and integrated density of regions of interest in the bioluminescence and fluorescence images. The difference was considered as statistically significant only when *P* < 0.05. The statistical analysis and data graphs were conducted by GraphPad Prism 5 (GraphPad Software Inc., San Diego, CA, USA).

## Results

### Characteristics of orthotopic neuroblastoma murine model

**Fig. 1:**
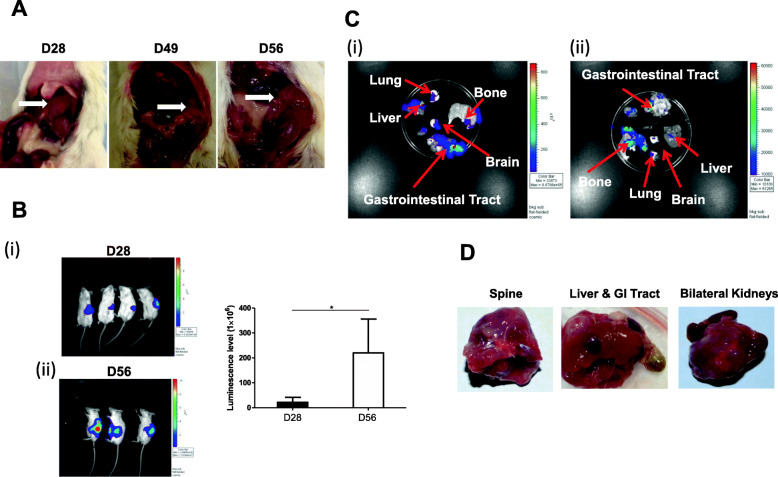
Tumor growth and metastasis of orthotopic neuroblastoma model. Tumor developed at adrenal inoculation site at Day 28, Day 49 and Day 56 after SK-N-LP cell transplantation, which could be directly observed in mice (**a**). Significant increase (*P* < 0.05) in bioluminescent detection of the tumor growth was demonstrated from Day 28 (*n* = 4) to (**b(i)**) and Day 56 (*n* = 3) in (**b (ii)**) in vivo by IVIS in vivo imaging system, respectively. Unpaired one-tailed t-test was used to compare bio-luminescence of the two groups. ^*^ indicates *P* = 0.0456 < 0.05. The color bar on right hand side showed the gradient of tumor activity from the highest (red) to the lowest (dark blue). Local and distant metastasis in brain, lung, liver, gut and bone was observed by detecting the bioluminescent signal, the representative result of one mouse was shown (**c(i)**). Involvement of bone and spine as major organs rather than liver and lung was observed in one mouse (**c (ii)**). Gastrointestinal tract, kidney, liver and lower spine encompassed by tumor could be shown in (**d**). The statistical analysis and data graphs were conducted by GraphPad Prism 5

Transplantation of human SK-N-LP cells into SCID-beige mice was demonstrated as being capable of generating stable orthotopic neuroblastoma which was validated by both in vivo imaging of detecting the bioluminescent signals tagged at SK-N-LP cells and by evaluating the gross tumors harvested from mice. The pronounced progression of tumor growth after the xenogeneic transplantation of human neuroblastoma cell line was shown in Fig. 1. Notable increase of tumor size was detected from Day 28, Day 49 to Day 56 post-inoculation (Fig. 1a). Rising intensity of photon emission was observed using Xenogen IVIS in vivo imaging from Day 28 (4 out of 4 mice) to Day 56 (3 out of 4 mice) (*P* = 0.0456 < 0.05) (Fig. 1b).

In accordance with the clinical manifestations, this neuroblastoma model demonstrated the characteristics of metastasis in multiple organs for all mice. Bone, brain, lung, liver, and gastrointestinal tract were affected. It was observed that liver, lung, gut and bone were the major organs invaded in almost all the mice (Fig. 1c**(i)**). In addition, our mice also exhibited variable clinical behaviors of metastasis observed in humans. In one of the mice, the major organs involved were bone (Fig. 1c**(ii)**) and spinal cord (Fig. 1d) rather than liver and lung. And this mouse developed severe paralysis of lower limbs and were clinically unable to crawl and the mouse received euthanasia by an overdose of pentobarbital before Day 56. In contrary to human scenario, gastrointestinal tract and kidney encompassed by tumor was commonly seen phenomenon in all the mice, and the adhesion of tumor to liver or lower spine appeared in one of the mice suggesting this neuroblastoma cell line SK-N-LP is highly invasive and may progress in diverse directions (Fig. 1d).

### hMSCs accelerated the initiation and growth of neuroblastoma

**Fig. 2:**
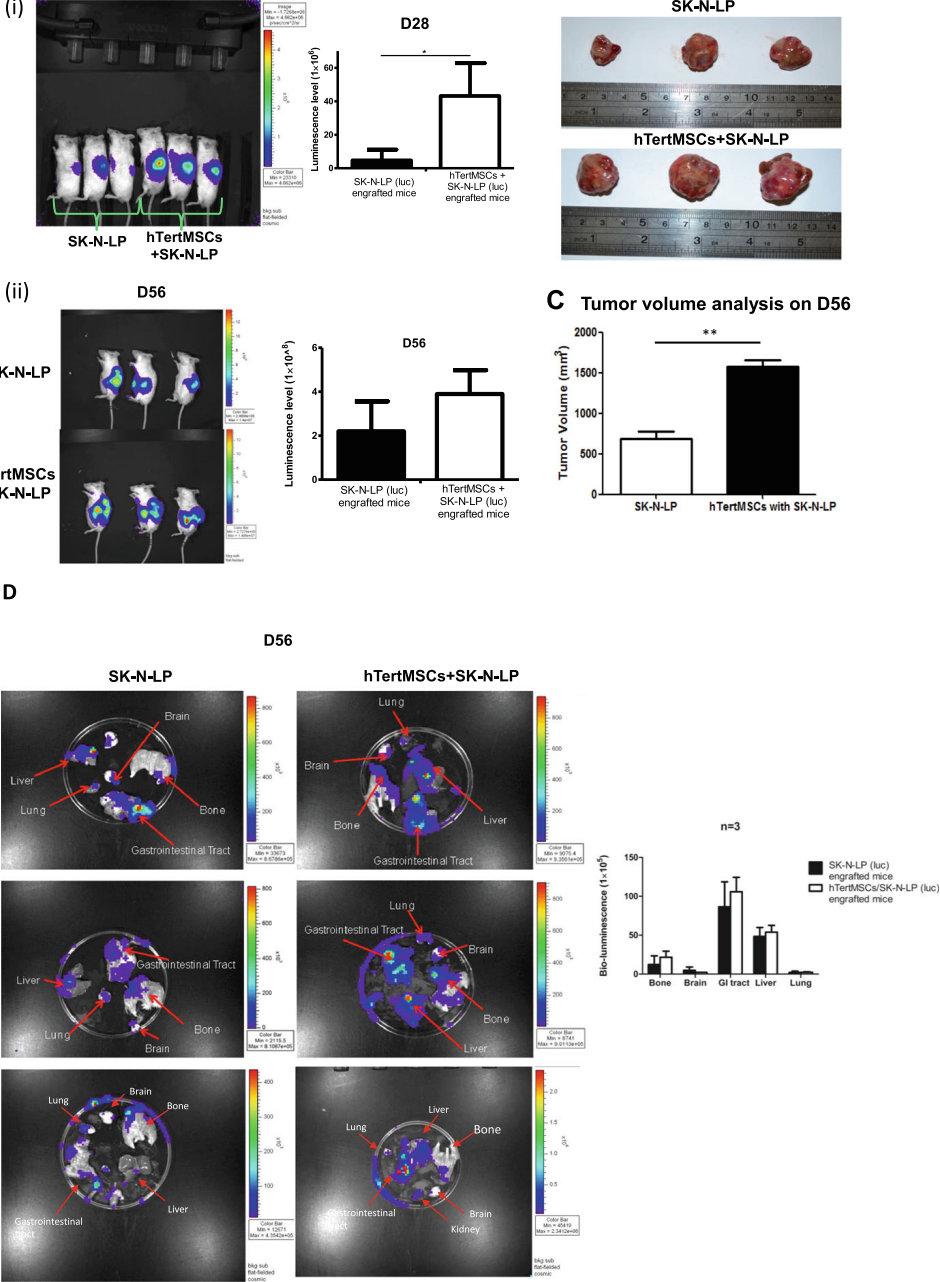
hMSCs enhanced tumor growth and apparently metastasis. Significant difference in tumor bioluminescence could be observed at Day 28 between the group of co-transplanted with hMSCs (*n* = 3) and SK-N-LP alone group (n = 3) detected by Xenogen IVIS 100 in vivo imaging and analyzed by unpaired one-tailed t-test, ^*^ indicates *P* = 0.01598 < 0.05 (**a(i)**). Difference in tumor bioluminescence was not statistically significant between 2 groups at Day 56, *P* = 0.05378 (n = 3) (**a (ii)**). Tumors were harvested (both groups) on Day 56 post-transplantation and the group with hMSCs and SK-N-LP co-transplantation showed significantly higher volume (**b**) and bigger tumor size (**c**) comparing to the SK-N-LP alone group. Quantified bio-luminescence was analyzed by unpaired one-tailed t-test, ^*^ indicates *P* < 0.05. Metastasis observed in harvested organs (lung, liver, gastrointestinal tract, brain and bone) from co-transplantation group was apparently higher than SK-N-LP alone group, although not statistically significant difference at Day 56 post-transplantation by detecting bioluminescent signal of SK-N-LP (representative results) (**d**). The statistical analysis and data graphs were conducted by GraphPad Prism 5

As early as the Day 28 post-transplantation, clear bioluminescent signals were detectable using Xenogen IVIS 100 imaging indicating the early formation of tumor in vivo. Compared to SK-N-LP only transplantation group, the bioluminescent signal was notably stronger in the group receiving the co-transplantation of hMSCs and SK-N-LP suggesting hMSCs may accelerate the in vivo initiation of neuroblastoma (*P* = 0.01598 < 0.05, *n* = 3) (Fig. 2a**(i)**). The difference in neuroblastoma progression was consistent for mice with 56 days of tumor implantation as confirmed by the calculation of harvested gross tumor volume, although statistically insignificant in bioluminescent signal (*P* = 0.05378, *n* = 3) (Fig. 2a**(ii)**). The volume and size of tumors harvested from co-transplantation group was significantly higher than these isolated from SK-N-LP alone group (*P* = 0.002 < 0.01, *n* = 3) (Fig. 2b, c). Above results supported that hMSCs promoted the early initiation and progression of neuroblastoma in vivo.

### hMSCs apparently promoted the metastasis of neuroblastoma

Since hMSCs indicated favorable impact on tumor growth, we further evaluated whether they could promote metastasis as well. At Day 56 post-inoculation, bioluminescent signals were analyzed using IVIS 100 imaging system. The bioluminescence in organs (brain, bone, liver, lung and gastrointestinal tract) from co-transplantation group was apparently greater than the SK-N-LP alone group, although no significant differences statistically (*n* = 3). Also, the bioluminescence was only weakly detectable in the brain in one of the mice in SK-N-LP group (Fig. 2d). Based on this observation, we postulated that hMSCs apparently enhance local or distant metastasis of neuroblastoma and the involved sites of metastasis might happen by chance.

### Recruitment of hMSCs towards primary tumor was partly CXCR4-dependent

**Fig. 3:**
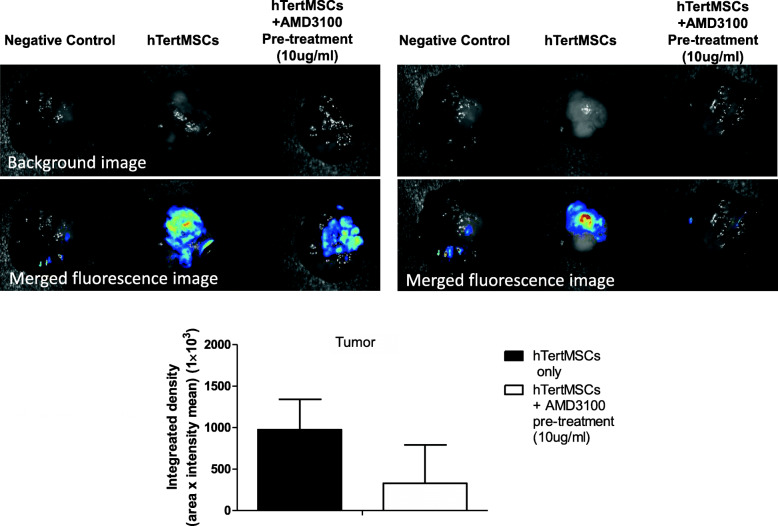
Recruitment of hMSCs towards primary tumor was CXCR4-dependent. CM-DiI-labeled hMSCs pre-treated with or without 10μg/ml AMD3100, the specific CXCR4 antagonist, were injected into mice (*n* = 4 for PBS control group and AMD3100 pre-treated hMSCs group) with neuroblastoma via tail vein at Day 48 after orthotopic tumor engraftment. By detecting the fluorescence signal by CRI in vivo imaging system, hMSCs were observed to be preferentially attracted by primary tumor which was consistently, (although not statistically significant *P* = 0.08, *n* = 3, representative results of 2 mice shown) inhibited by AMD3100. Data was analyzed by unpaired one-tailed t-test. The data graphs were conducted by GraphPad Prism 5

CM-DiI-labeled hMSCs pre-treated with or without AMD3100 were injected into mice with neuroblastoma (*n* = 4), the fluorescence signal was detected using in vivo imaging. We observed that the fluorescence signal of CM-DiI could be detected at primary tumor site indicating hMSCs were attracted by neuroblastoma. The signal intensity was consistently reduced when hMSCs were pre-treated with CXCR-4 inhibitor AMD3100, although statistically insignificant [*P* = 0.08 between AMD3100 treated and PBS control groups, *n* = 3 (one mouse from each group died before the day of harvesting organ)]. These findings suggested that this trafficking process was partly inhibited by AMD3100 (Fig. 3). These observations revealed that hMSCs might preferentially migrate towards primary tumor and was partly CXCR4-dependent.

### hMSCs could be recruited to the metastatic loci of neuroblastoma

**Fig. 4:**
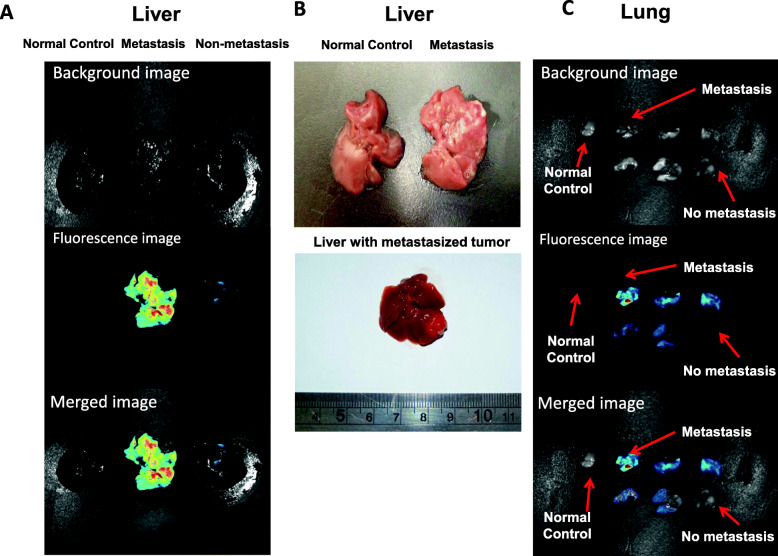
Selective recruitment of hMSCs by metastatic loci. The recruitment of hMSCs towards liver and lung with or without metastatic disease was compared and detected by fluorescence signal by harvesting the mice organs on Day 56 after orthotopic tumor engraftment. The neuroblastoma metastasis in liver (n = 3, representative results) and lung (n = 3) was detectable by bio-luminescence using in vivo imaging system, (**a, c**). Liver metastatic loci could be directly observed in gross tumors ex vivo, representative results (**b**). hMSCs could be selectively attracted by liver (**a**) and lung (**c**) invaded by neuroblastoma cells. None could be detected in the liver (**a**) and lung (**c**) without tumor metastasis, representative results

**Fig. 5:**
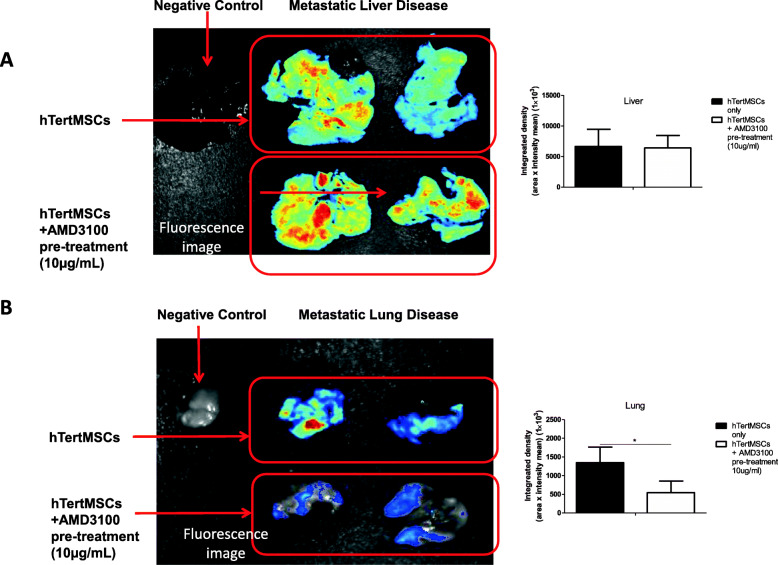
Recruitment of hMSCs by metastatic loci could not be completely inhibited by CXCR4 specific antagonist AMD3100. We traced the hMSCs trafficking in vivo by detecting the fluorescence signal of CM-DiI pre-labeled hMSCs in freshly harvested livers and lungs in Day 56 after orthotopic tumor engraftment. Fluorescence image quantified data was analyzed by un-paired one-tailed t-test. 10μg/ml AMD3100 pre-treatment could not significantly inhibit the recruitment of hMSCs towards the liver with metastatic disease (*P* = 0.4465, n = 3, representative results of 2 mice shown) (**a**), but could significantly block the recruitment towards the lung invaded with tumor cells, ^*^indicates *P* = 0.04548 < 0.05 (n = 3, representative results of 2 mice shown) (**b**). The statistical analysis and data graphs were conducted by GraphPad Prism 5

In addition to the homing towards primary tumor, CM-DiI labeled hMSCs were also detected at metastatic loci of neuroblastoma as shown in Fig. 4; however, the fluorescence signal of hMSCs were not detected in unaffected liver (Fig. 4a) and lung using in vivo imaging (Fig. 4c). Such findings indicated that hMSCs might be selectively recruited by the metastatic loci of neuroblastoma. In contrary to the potent suppressive influence on the trafficking towards the primary tumor site, AMD3100 treatment could not completely inhibit the recruitment of hMSCs to the metastatic loci of neuroblastoma (Fig. 5). AMD3100 treatment significantly inhibited the recruitment of hMSCs to lung (*P* = 0.04548 < 0.05 between AMD3100 treated and PBS control groups, *n* = 3) **(**Fig. 5b**)**, but not to liver (*P* = 0.4465 between AMD3100 treated and PBS control groups, *n* = 3) (Fig. 5a).

## Discussion

In our present study, we utilized an orthotopic murine model of neuroblastoma to mimick the neuroblastoma microenvironment in vivo. Adrenal gland is the most common primary site of neuroblastoma and implanting xenografts of human neuroblastoma cells into the adrenal gland area creating a more relevant microenvironment setting compared to the subcutaneously implanted in vivo tumor model. This model also allows us to evaluate the metastatic potential of neuroblastoma cells in vivo. In our model, bioluminescence gene luciferase was used to label transplanted tumor cells, which can be utilized to monitor the early growth of tumor in vivo non-invasively. This model is free of apparent auto-fluorescence background since luciferase cannot be produced naturally by mice. Thus, this orthotopic model provides a better platform for investigating the underlying tumorigenic pathophysiology. In addition, it can also help to evaluate the efficacy of novel anti-cancer therapies.

We further explored the effects of hMSCs on the initiation and progression of human neuroblastoma. Until now, the exact impact of MSCs on tumor growth and progression in vivo is still controversial. The favorable effects of hMSCs have been reported in the tumorigenesis of ovarian cancer [19], colorectal carcinoma [20], breast cancer [8] and pancreatic cancer [9]. The potential underlying mechanisms include the supportive effects of MSCs on cancer growth and metastasis. MSCs can enhance tumor growth via constituting the cancer microenvironment, enhancing neovascularization, producing growth factors and exerting immunosuppressive effects. In addition, MSCs enhance cancer metastasis through releasing soluble factors such as chemokine SDF-1, IL-6 and CCL5. They impact on cancer metastasis through releasing soluble factors such as chemokine SDF-1, IL-6 and CCL5 [4–6]. Moreover, MSCs protect cancer cell survival from cytotoxicity of anti-cancer reagents [21]. However, MSCs also exerted negative effects on the growth of colon carcinoma [22], Kaposi’s carcinoma [23], glioma [12] and hepatoma model [11]. Conflict findings were reported even in same type of cancer or study including neuroblastoma [14, 15, 17, 24–27]. To date, the exact reasons underlying these controversial effects remain largely unknown. It is potentially related to the specific histological types of cancer, experimental model and research design, different in vitro culture conditions and the dosage of cell inoculated. Furthermore, such effects could also be closely related to tumor-specific background and in certain scenarios even be cell-line specific. Majority of studies observed the supportive effects of co-transplanted MSCs on cancers using excessive number of MSCs than cancer cells or at least with equal number. Our study validated that lower dosage of MSCs co-injection with neuroblastoma cells (hMSCs: NB cells = 1:2 as 1 × 10^5^ hMSCs: 2 × 10^5^ neuroblastoma cells) was enough to enhance tumor growth and metastasis. However, much lower dosage of hMSCs (10^2^ hMSCs to 10^4^ cancer cells, i.e. hMSCs: cancer cells = 1:100) was found to induce tumor rejection [28].

The data from current research focusing on the interaction between MSCs and neuroblastoma is very limited. It was revealed that MSCs could protect neuroblastoma from oxidative stress in vitro [29]. IL-6 produced by MSCs was reported to participate in promoting survival of neuroblastoma cells and bone metastasis [30]. In addition, SDF-1/CXCR4 axis plays a pivotal role in growth, progression and metastasis modulation in diverse kinds of cancers including head and neck cancer, pancreatic cancer and lung cancer. It was suggested that SDF-1/CXCR4 axis could promote the dissemination of cancer cells towards sites highly secreting SDF-1. SDF-1 binds to the cognate receptor CXCR4 expressed on cancer cells including neuroblastoma [31–33]. Our previous in vitro study demonstrated that MSCs benefit the metastasis of neuroblastoma via the secretion of SDF-1 [16]. It was also reported that MSCs secretome could modulate CXCR4 expression and invasion to the bone marrow of neuroblastoma in vitro [34]. Despite the above insight that we obtained from various researches, the exact role of MSCs in neuroblastoma development has yet to be clearly defined and majority of data require further validation by in vivo experiments*.* In this study, we demonstrated that hMSCs indeed exerted tumorigenic effects on neuroblastoma in vivo. In early period of post-inoculation, mice co-transplanted with hMSCs and SK-N-LP showed stronger tumor signals compared to the mice injected with SK-N-LP alone. Such phenomenon was further verified by evaluation of gross tumor volume after longer time of inoculation. The facilitative effect of hMSCs on neuroblastoma’s metastasis was also studied. We observed that compared to SK-N-LP group, hMSCs co-transplantation apparently accelerate the metastasis since the mice in this group developed metastasis with apparently higher bioluminescent signals in all organs studied.

Based on the above results, we further explored whether hMSCs could be a therapeutic target to eradicate tumors from the sanctuary microenvironment. To achieve this goal, we investigated the migration of hMSCs in mice bearing neuroblastoma and investigated the potential modifier involved in this whole process. It has been extensively reported that SDF-1/CXCR4 axis is actively involved in the homing of MSCs to injured tissues and thereafter exert biological immunomodulatory and regeneration effects. MSCs were also found to have the propensity of being guided towards tumors. However, unlike the advanced understanding of MSCs migration to injured tissues, the mechanism responsible for homing of MSCs towards tumors is just starting to be unfolded and it has not been adequately explored especially in the setting of neuroblastoma. Whether SDF-1/CXCR4 axis is a vital modifier in MSCs homing towards neuroblastoma, like it is described in the trafficking towards injured tissues remains uncertain. We found that hMSCs could preferentially migrate to neuroblastoma. Importantly, we also demonstrated that such migratory drive was in a CXCR4-dependent manner. Pretreatment with AMD3100, the specific antagonist of CXCR4, consistently reduced the homing of hMSCs towards primary tumor. The evidence supporting this concept came from the phenomenon that hMSCs signal was not detected in liver or lung without metastatic disease. Moreover, supplementing the early studies, we observed that systemically infused hMSCs could also be attracted to the metastatic loci other than the primary tumor.

To the best of our knowledge, few studies demonstrated the preferential homing of MSCs towards metastatic loci. One study reported intravenously injected MSCs could be guided towards primary tumor and lung metastatic sites. However, they established the tumor model using subcutaneous inoculation and the lung metastasis was induced separately through intravenous injection of tumor cells. After infusing the MSCs systemically 4 days post-injection of cancer cells, higher signal intensity and longer retention of MSCs were noted at lung than normal control and thus suggested the specific recruitment of MSCs by metastatic lesions [35]. This can be due to trapping of cancer cells in the lung tissue via the “first-pass effect” during venous return to the right heart and then the lung. In our orthotopic tumor model,, multiple pulmonary and extra-pulmonary metastatic diseases originated from the primary tumor were detected. This simulated the actual clinical scenario. Using this clinically relevant model, we provided convincing evidence for the tumor tropism of MSCs. Firstly, it was demonstrated that intravenously injected hMSCs could preferentially home towards both the primary tumor site and the multiple metastatic loci. Through labeling the hMSCs and tumor cells, we can directly observe the preferential migration of hMSCs to organs invaded, whereas, no hMSCs were detected in normal tissues. This property of tropism provides a promising cue for targeting the microenvironment in high risk metastatic neuroblastoma.

Interestingly, albeit consistent inhibitory effects on hMSCs trafficking towards primary tumor and lung, AMD3100 pretreatment failed to show similar impact on the recruitment of hMSCs in liver. The mechanism underlying this paradoxical phenomenon warrants further detailed investigations. We hypothesize several possible mechanisms underlying this phenomenon. The first potential reason is the level of SDF-1 was much higher at metastatic loci than primary site [36]. The inhibition of MSCs homing towards metastatic loci may require much higher dosage of AMD3100 than used in blocking the migration of MSCs towards primary site as AMD3100 is a competitive inhibitor of CXCR4 [37]. In support of this, using high-density tissue microarrays, a large cohort study of more than 600 human prostate carcinoma specimens indicated higher SDF-1 was expressed by metastatic lesions when compared to primary tumor sites [38]. In addition, CXCR7, the other receptor of SDF-1, was reported to express in cancer cells and associated with tumorigenesis and metastasis [36, 39, 40]. In our previous study, both CXCR4 and CXCR7 were found to express in neuroblastoma cell lines. CXCR7 was involved in increasing neuroblastoma migration via alternative receptor of SDF-1 in the absence of CXCR4, but has no role in regulating normal cell migration and adhesion [16]. However, the exact role of CXCR7 in guiding the homing of MSCs towards tumor metastatic loci deserves further study. Finally, the potential variation in microenvironment between primary tumors and metastatic lesions may result in different profiles of released chemokines. The metastatic lesions might trigger different tissue injury signals and involved other non-SDF-1 related pathways.

There were some limitations in current study which could not ascertain the effects of hMSCs on early metastasis of neuroblastoma. Therefore further in vivo studies are still required. As the mice we used were immune-deficient, we were unable to investigate the role of immune system during the tumor progression and metastasis. Also, we could not completely rule out the possibility that neuroblastoma cells may accidentally travel through blood circulation to distal organs during initial inoculation, causing false-positive metastasis. Using more mice for harvesting organs regularly at earlier time point might help in resolving this technical limitation. There are also uncertainties regarding the hMSCs homing which deserves further investigation. The sequence in which intravenously infused hMSCs migrate towards primary tumor or metastatic loci also remains unknown. Moreover, one notable phenomenon that AMD3100 pre-treatment could not completely inhibit the homing of hMSCs towards metastatic loci requires further verification as our study couldn’t determine whether the incomplete inhibition was due to insufficient dosage or the presence of alternative pathway(s) in mediating hMSCs homing. In addition, albeit useful, it was observed that the bioluminescence imaging underestimated the tumor burden at longer time period.

## Conclusions

**Fig. 6:**
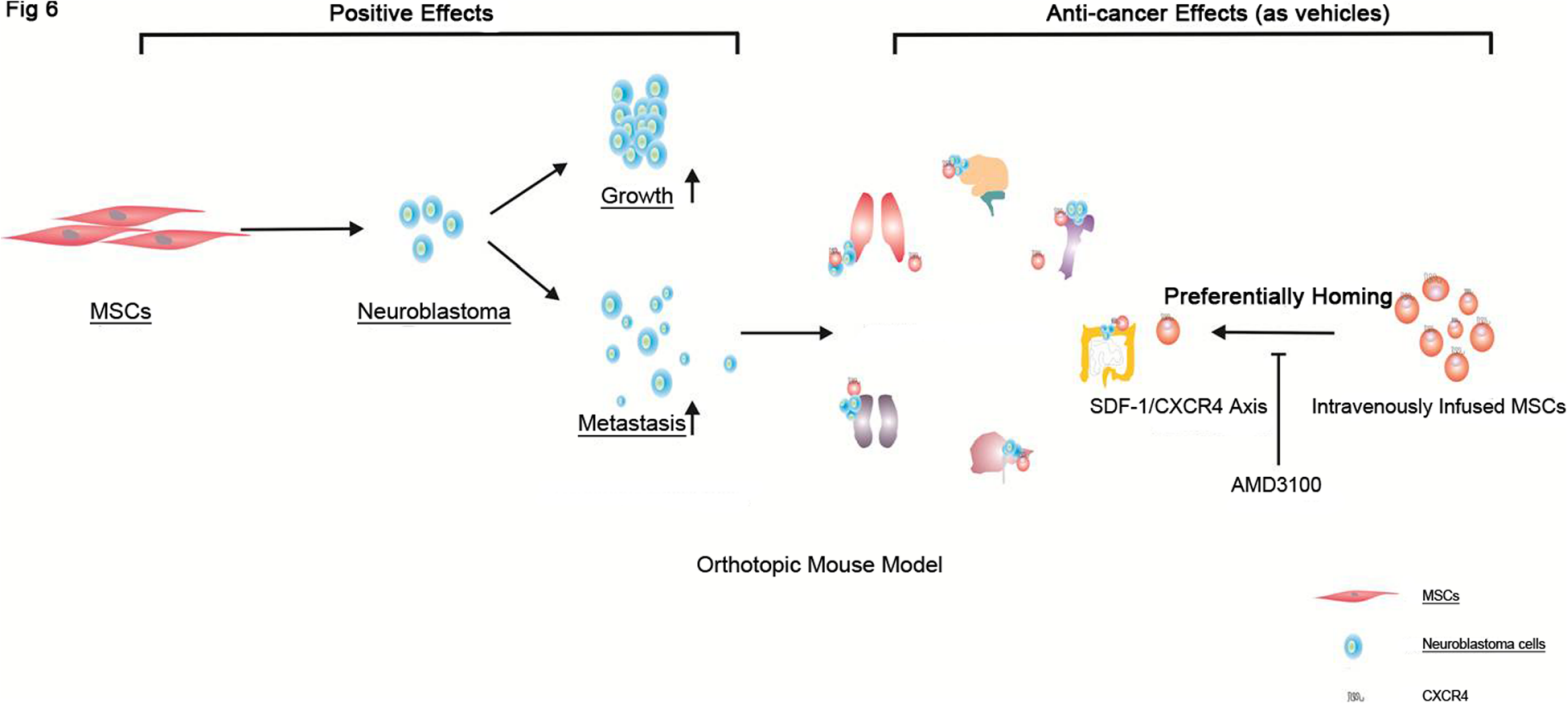
The “positive” and “anti-cancer” effects of MSCs on neuroblastoma. MSCs exhibited potent effects on accelerating the potency of tumor formation, growth and metastasis in vivo. On the other hand, intravenous infused MSCs could be recruited by both primary tumor and metastatic loci, which could be reduced when MSCs were pre-treated with SDF-1 blocker, AMD3100. The preferentially homing propensity of MSCs provides new therapeutic insights in utilizing bio-engineered MSCs as vehicles for targeted anti-cancer therapy against advanced cancers

In conclusion, a stable xenogeneic orthotopic neuroblastoma model wasestablished using adrenal injection with human neuroblastoma cells and was validated to be able to grow and metastasize to various distant sites. Bioluminescence detection could be used to monitor the tumor growth especially during the early initiation phase of tumor in vivo. hMSCs exhibited potent effects on accelerating the tumor formation, growth and metastasis. These results provide the evidence of using MSCs as the promising therapeutic target in the future clinical applications. Furthermore, the preferential homing propensity of MSCs towards both primary tumor and metastatic loci could also bring the new therapeutic insights in utilizing bio-engineered MSCs as vehicles for targeted anti-cancer therapy especially against the advanced diseases (Fig. 6).

## Supplementary Information

**Additional file 1: Supplementary Figure S1.** Experimental design. (**A**) Experimental design for exploring the effects of hMSCs on the growth and metastasis of neuroblastoma. The difference of growth and metastasis of tumor between SK-N-LP group (*n* = 4) and hMSCs co-transplantation group (*n* = 4) was compared by Xenogen IVIS 100 in vivo imaging and analyzed by tumor volume evaluation at Day 28 and Day 56 after cell transplantation, respectively. (**B**) Experimental design for exploring the tumor tropism property of hMSCs towards primary tumor and metastatic loci. hMSCs pre-treated with PBS (hMSCs group, *n* = 4) or specific CXCR4 antagonist AMD3100 (hMSCs+AMD3100 group, *n* = 4) were intravenously injected into mice with implanted neuroblastoma 48 days post-surgery via tail vein, respectively. The recruitment of hMSCs towards primary tumor or metastatic loci was observed by CRI Maestro™ in vivo imaging system.

## Data Availability

The data sets analyzed during the current study are available from the corresponding author, Prof. G.C.F Chan, on reasonable request.

## Abbreviations

hMSCs: Human mesenchymal stem cells
SDF-1: Stromal cell derived factor-1
CXCR4: C-X-C chemokine receptor4
MSCs: Mesenchymal stem cells
DMEM: Dulbecco’s Modified Eagles Medium
FBS: fetal bovine serum
PBS: phosphate buffered saline
IVIS 100: In vivo imaging system Xenogen IVIS100
