# Supplementary material for: Mesenchymal stem cells accelerated growth and metastasis of neuroblastoma and preferentially homed towards both primary and metastatic loci in orthotopic neuroblastoma model
Source: BMC Cancer. 2021 Apr 10;21:393. doi: 10.1186/s12885-021-08090-2 (PMC8035760; doi:10.1186/s12885-021-08090-2)
Supplement: Supplementary file 1 — Additional file 1: Supplementary Figure S1. Experimental design. (A) Experimental design for exploring the effects of hMSCs on the growth and metastasis of neuroblastoma. The difference of growth and metastasis of tumor between SK-N-LP group (n = 4) and hMSCs co-transplantation group (n = 4) was compared by Xenogen IVIS 100 in vivo imaging and analyzed by tumor volume evaluation at Day 28 and Day 56 after cell transplantation, respectively. (B) Experimental design for exploring the tumor tropism property of hMSCs towards primary tumor and metastatic loci. hMSCs pre-treated with PBS (hMSCs group, n = 4) or specific CXCR4 antagonist AMD3100 (hMSCs+AMD3100 group, n = 4) were intravenously injected into mice with implanted neuroblastoma 48 days post-surgery via tail vein, respectively. The recruitment of hMSCs towards primary tumor or metastatic loci was observed by CRI Maestro™ in vivo imaging system. [file 12885_2021_8090_MOESM1_ESM.ppt]

## Slide 1
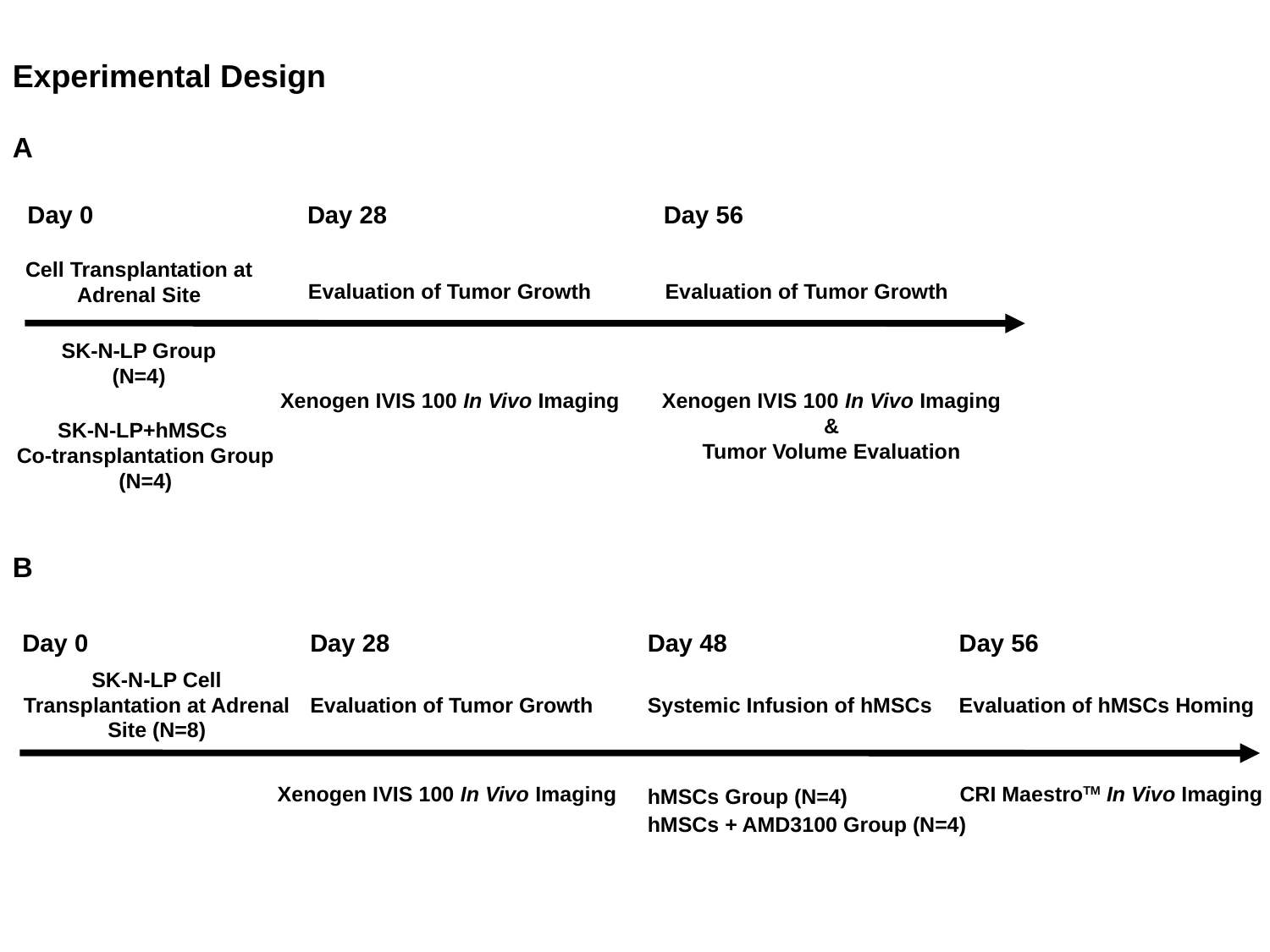

Experimental Design
A
Day 0
Day 28
Day 56
Cell Transplantation at Adrenal Site
Evaluation of Tumor Growth
Evaluation of Tumor Growth
SK-N-LP Group
(N=4)
Xenogen IVIS 100 In Vivo Imaging
Xenogen IVIS 100 In Vivo Imaging
&
Tumor Volume Evaluation
SK-N-LP+hMSCs
Co-transplantation Group
(N=4)
B
Day 0
Day 28
Day 48
Day 56
SK-N-LP Cell Transplantation at Adrenal Site (N=8)
Evaluation of Tumor Growth
Systemic Infusion of hMSCs
Evaluation of hMSCs Homing
Xenogen IVIS 100 In Vivo Imaging
hMSCs Group (N=4)
hMSCs + AMD3100 Group (N=4)
CRI MaestroTM In Vivo Imaging
